# Quality of Life and Its Related Factors in Chinese Unemployed People: A Population-Based Cross-Sectional Study

**DOI:** 10.3390/ijerph13080797

**Published:** 2016-08-08

**Authors:** Xiaoshi Yang, Lutian Yao, Hui Wu, Yang Wang, Li Liu, Jiana Wang, Lie Wang

**Affiliations:** 1 Department of Social Medicine, School of Public Health, China Medical University, Shenyang 110122, China; yangxs@mail.cmu.edu.cn (X.Y.); wuhui@mail.cmu.edu.cn (H.W.); yangwang@cmu.edu.cn (Y.W.); liul@mail.cmu.edu.cn (L.L.); jiana0818@163.com (J.W.); 2 Department of Surgery, First Affiliated Hospital of China Medical University, Shenyang 110001, China; yaolutian@gmail.com

**Keywords:** QOL, the unemployed, coping, self-efficacy, PCS, MCS

## Abstract

With the global economic crisis and industrial restructuring, the unemployed are suffering from job loss-related stress and loss of income, which is believed to impair their mental and physical health, while coping and self-efficacy could combat the adverse effects of unemployment on health. Thus, this study aims to describe quality of life (QOL) among unemployed Chinese people and explore the associated factors. A cross-sectional study was conducted by convenience sampling, composed of 1825 unemployed people, from January 2011 to September 2011. Questionnaires pertaining to demographic characteristics, the 36-item Short-Form Health Survey (SF-36), the abbreviated version of the Cope Inventory (Brief COPE) and self-efficacy scales were used to collect information from unemployed people in the eastern, central, and western regions of China. Hierarchical multiple regression analysis was performed to explore the related factors of QOL. A structural equation model (SEM) was used to test the relations among coping, self-efficacy, and QOL. Mental QOL was significantly lower than physical QOL in Chinese unemployed people. Coping had significant effects on both physical component summary (PCS) and mental component summary (MCS), while self-efficacy played the mediating role in the association between Coping and QOL. Unemployed Chinese people’s mental QOL was disrupted more seriously than their physical QOL. An increase in coping could improve QOL by promoting better management of issues brought about by unemployment. In addition, self-efficacy has the ability to reduce the impact of unemployment on QOL, through the mediating path of coping on QOL. This study highlights the need of coping skills training and self-efficacy enhancement for better management of unemployment in order to improve QOL and well-being.

## 1. Introduction

With the global economic crisis and industrial restructuring, unemployment problem has become an important social issue in China. The 2011 annual statistical bulletin of the development of Human Resources and Social Security reported that the urban registered unemployment rate was 4.1% in 2011. The ratio between the registered unemployed and the employed was 1:13.8. When the hidden number of the unemployed people was taken into account, the ratio between the unemployed and the employed would be 1:10.1 [1]. Obviously, unemployment is a significant stressor, which threatens individuals’ social welfare and professional life. Most researchers have found that unemployment primarily results in increased stress levels [2,3]. It also presents a tremendous economic challenge as a result of a decreased work force, which may have an obvious effect on mental health, and could consequently increase the risks of both somatic and mental disorders [4,5]. Unemployed individuals have a higher prevalence of depression, dermatitis, headaches, and ulcers as compared to the employed [6]. In line with previous findings, results showed that unemployment resulted in the destruction of self-efficacy [7], which had harmful effects on their health. It was also found that attempted suicide and suicide were strongly related with unemployment [8,9,10,11]. Unemployment has tremendous detrimental effects on physical and mental quality of life (QOL) and leads to lower levels of life satisfaction and well-being [12].

Recent publications indicated that the health of the unemployed was associated with household income, duration of unemployment, and the negative effects of chronic disease [13,14,15]. Some, but not all, of the difference can be explained by demographic differences, leading some researchers to conclude that the unemployed are exposed to higher levels of stress factors that affect health [16,17]. Additionally, material support, social support, and other contextual factors, such as the macroeconomic environment, are highly associated with the unemployed health [18,19]. However, nowadays in China, the policy support for the unemployed is very limited except for unemployment insurance and social relief for the poor. Since the macroeconomic environment is unchangeable, the unemployed people’s health in this study was studied mainly from the view of the individual levels to understand what strategies should be given to unemployed individuals in order to promote their health.

The transactional stress model theory proposed by Lazarus and Folkman 1984 [20], indicated that an individual’s reaction to stress could be influenced by the process of coping with an event that has been appraised as stressful. Coping with unemployment refers to an individual’s cognitive and behavioral efforts to manage the demands as a result of their situation, responding to stressors by the appropriate use of adaptive coping resources. The process of coping is directed towards influencing the impact of such events on an individual’s physical, social, and emotional function. Individuals with adaptive coping could positively deal with the bad effects of unemployment and protect their health. Individuals with a strong coping capability can avoid breakdowns [21] and, thus, alleviate mental health problems and promote physical health, while maladaptive coping, like avoidance and denial, was found to be associated with anxiety and depression [22,23]. Numerous researchers have suggested that maladaptive coping may result in the adverse effect of the unemployment on overall health [24,25].

The transactional stress model also indicates that, appraisals of situations and coping are affected by personal resources such as perceived control, self-efficacy, personality, and social resources [26]. Self-efficacy has yielded various findings in regards to individuals’ health, which is the positive capacity of an individual to manage a stressful situation, serving as a self-regulatory function. It influences their own cognition, affects actions, and shapes their environment, accordingly, minimizing symptoms of stress [27]. Coping may also affect the individuals’ self-efficacy to cope with stress when engaging in a task and the degree of accomplishment they realize. The positive effects of self-efficacy have been verified in various fields, including smoking control, pain control, coping with chronic disease and health [28,29]. In one study of cancer patients which showed the importance of coping for adjustment to stress, self-efficacy partially mediated the positive relations of coping to other variables [30]. Likewise, self-efficacy may help to minimize the adverse effects of maladaptive coping to unemployment, and promote individuals to deal with stressful situations and it has been widely applied as one of the psychotherapy interventions to the unemployed [31]. Coping not only affects QOL directly, but also indirectly through the mediating path of self-efficacy influencing QOL. Following this line of reasoning, it is hypothesized that (1) demographic characteristics, such as the household income, duration of unemployment, and chronic disease, could affect QOL of the unemployed; (2) there is a positive association between coping and QOL; and (3) self-efficacy plays a mediating role on the association between coping and QOL. However, few studies have been reported about Chinese unemployed individuals. Thus, the aim of this study was to assess the QOL of the Chinese unemployed individuals, explore the associated factors of QOL, and examine the relationships of coping, self-efficacy, and QOL. The findings from this study may be used to provide potential management strategies to attenuate the negative effects of unemployment on the QOL.

## 2. Methods

### 2.1. Study Design and Sample

A cross-sectional study was conducted from January 2011 to September 2011. The sample employed in this study consisted of 2500 unemployed individuals. Multi-stage convenience sampling was adopted in this study. During the first stage, a total of 11 provinces and one municipality in the eastern, central, and western regions of China were selected (four provinces from the east China, four provinces from the western region, and three other provinces, along with the municipality of Beijing, in the central region of China, were selected). During the second stage, two cities were randomly extracted from each province (total of 22 cities and one municipality in the eastern, central, and western regions of China were selected). During the third stage, the unemployed people based on the criteria of North America [32], by convenience sampling, aged 18 years and older, who are jobless, and have been actively looking for work within the past four weeks, from four to five large labor markets (contains at least 300 registered unemployed people) of each city were enrolled in this study.

### 2.2. Ethics Statement

If the selected persons agreed to participate, they were asked to sign consent forms. All of the participants were well-informed of the contents and aim of the questionnaire. After obtaining written informed consent about the conduct of the survey, the unemployed were interviewed face-to-face by trained college students from China Medical University. The procedures followed were in accordance with, and approved by, the ethical standards of the Committee on Human Experimentation of China Medical University. All subjects gave their informed consent for inclusion before they participated in the study. The study was conducted in accordance with the Declaration of Helsinki, and the protocol was approved by the Ethics Committee of China Medical University (CMU1210400026).

Among the 2500 unemployed individuals, 456 declined to participate, resulting in an 81.76% consent rate. Eighty-six were excluded because they dropped out of the study, and 133 were excluded because of missing values exceeding 20% in the questionnaire. The characteristics (sex, age, educational level, and household monthly income) of the remaining 1825 respondents included in the analysis were similar to those of the 542 individuals who were excluded. Of all the participants, 1825 individuals took part in and answered the questionnaire effectively, resulting in a valid response rate of 73.0%.

### 2.3. Demographic Characteristics

Demographic characteristics included age (<25, 25–39, >39 years older), gender, marital status (married/others, including unmarried/widowed/divorced/separated), educational level, household monthly income, and chronic diseases. Educational level was categorized as “≤ senior high school or below”, and “> senior high school”. Household monthly income (RMB) was classified as “≤1000 Yuan”, “1001–1500 Yuan”, “1501–2000 Yuan”, or “>2000 Yuan”. “Chronic diseases” was defined as “yes” if any common chronic disease (e.g., hypertension, cardiovascular disease, diabetes, and arthritis) had ever been diagnosed. Unemployed time was categorized as “1–2 months”, “3–5 months”, “6–11 months”, and “≥12 months”.

### 2.4. QOL

The 36-item Short-Form Health Survey (SF-36) [33,34], which has been widely used for different kinds of people in China, was applied to assess QOL among the unemployed people. The SF-36 consists of 36 items that measure eight different dimensions of health: physical function, role limitations related to physical problems, bodily pain, general health perception, vitality, social functioning, role limitations due to emotional problems and mental health, which can be categorized into the physical component summary (PCS) and mental component summary (MCS). The health concepts are described by a range from 0 to 100, with higher scores indicating better health. The Cronbach’s alpha coefficient was 0.8783 for this study.

### 2.5. Coping

Coping was evaluated by means of an abbreviated version of the Cope Inventory (Brief COPE) [35]. The Brief COPE consists of 28 items that measure 14 domains of coping strategies: self-distraction, active coping, denial, substance use, use of emotional support, use of instrumental support, behavioural disengagement, venting, positive reframing, planning, humour, acceptance, religion, and self-blame, which can be categorized into adaptive coping (active coping, planning, positive reframing, acceptance, humor, and seeking emotional and instrumental social support) and maladaptive coping (denial, venting, substance use, self-blaming, self-distraction, religion, and behavioral disengagement). Responses are rated on a four-point scale ranging from 1 (have not been doing this at all) to 4 (have been doing this a lot). The Cronbach’s alpha coefficient was 0.7967 in this study.

### 2.6. Self-Efficacy

Self-efficacy was measured with an adapted General Self-efficacy Scale by Song Zhaoli [36], which was evolved from the English version of the Generalized General Self-efficacy Scale [37,38]. It included 12 items rated on a five-point scale and the responses range from strong disagreement (1) to strong agreement (5), with higher scores indicating higher levels of self-efficacy. The scale provides an overall summative score and has been shown to be of high reliability (Cronbach’s α = 0.7558) and of good validity. For validity, the correlation coefficient of the 12 items and total scale score is between 0.60 and 0.77. In factor analysis, one factor was extracted and the explanatory variance is 40.11%, showing that it has good construct validity.

### 2.7. Statistical Analysis

The comparisons of QOL in categorical variables were evaluated using t-test and one-way analysis of variance (ANOVA). Pearson’s correlation coefficient was used to determine the relationship of QOL, coping and self-efficacy. Hierarchical multiple regression (HMR) analysis was conducted to test the incremental variance of any given set of independent variables. The unemployed individuals’ QOL scores were used as dependent variables. The independent variables were entered in the following steps: Step 1: demographic characteristics; Step 2: coping; and Step 3: self-efficacy. The analysis proceeded in stages by successively inputting several blocks of independent variables in the regression model. Blocks of variables entered in later stages were, thus, tested for their extra contribution. The relative importance of the variables retained in the final multiple regression models contributed to the explained variance of the QOL, which was represented as the standardized β [39]. Fit of the model was assessed according to the R^2^-value. Structural equation modeling (SEM) was also used to test the set of hypothesized relations among variables. Sobel tests were used in order to confirm the mediating role of self-efficacy in the association of coping and QOL. In the SEM model, QOL (PCS and MCS) were modeled as dependent variables, coping (adaptive coping and maladaptive coping) as independent variables, and self-efficacy as a mediator. The indicators included the χ^2^/df, goodness of fit index (GFI), comparative fix index (CFI), Tucker-Lewis index (TLI), and root mean square error of approximation (RMSEA), and were used as the criteria to assess the structural model. The adequate fit of the model was evaluated by the following indicators: χ^2^/df < 5, GFI, CFI, TLI > 0.90, and RMSEA < 0.08. If there was a reduction in the size of direct path coefficients of coping on QOL, or a disappearance of statistical significance when the mediator was added in model, the possibility of mediation was speculated. Then, the bootstrapping strategies were used to examine the mediating roles (a × b product) of self-efficacy on the associations of coping with QOL [40]. Bootstrapping, an increasingly popular non-parametric method of testing mediation effect, provides a powerful and reasonable method of obtaining confidence intervals (CI) for mediation effects under most conditions. The bootstrap estimate was based on 5000 bootstrap samples. A bias-corrected and accelerated 95% CI (BCa 95% CI) for each a × b product was investigated. A BCa 95% CI, excluding 0, indicates a significant mediating role. Statistical analysis was performed using the Statistical Package for Social Science Version 17.0 (SPSS Inc., Chicago, IL, USA) and Amos 6.0 (SPSS Inc., Chicago, IL, USA), and a two-tailed probability value of less than 0.05 was considered to indicate statistical significance.

## 3. Results

### 3.1. Description of the Demographic Characteristics

The basic characteristics of the participants are provided in Table 1. Of the participants, 1022 (56.0%) were men and 803 (44.0%) were women. The mean age was 34.9 years old (ranging from 18 to 65 years old). About two thirds (2/3) of the unemployed had the educational level of lower or equal to senior high school, and 58.79% of the unemployed were currently married. In this study, 22.85% and 21.42% of the unemployed people had a monthly household income level less than 1000 yuan (RMB) and 1001–1500 Yuan (RMB), respectively. More than half of the unemployed suffered from chronic diseases. Approximately half of the individuals lost their jobs at least six months previous to this study.

### 3.2. Description of QOL

The QOL scores of this study are listed in Table 2. The PCS mean scores for the female unemployed were significantly lower than the scores of the male unemployed, and the unemployed with the educational level of no higher than senior high school, or those that were married, suffered lower levels of PCS. Those unemployed who were less than 25 years old reported lower levels of both PCS and MCS than that of other groups. The greater the household monthly income, the higher the scores of QOL reported by the Chinese unemployed. The unemployed with chronic diseases exerted lower scores of PCS and MCS than those without chronic diseases. The unemployed with a longer time of unemployment, reported lower QOL. Moreover, mental QOL was significantly lower than physical QOL of the Chinese unemployed people.

Physical and mental QOL were significantly worse in individuals with low educational levels or who were married, and among persons with chronic diseases. Physical health was also significantly worse in women or participants with the ages of 30 and above. Respondents with a longer period of unemployment had a lower level of physical and mental health than those who had been unemployed for a shorter period. Adaptive coping and self-efficacy were significantly and positively correlated with PCS and MCS, while maladaptive coping was negatively correlated with PCS and MCS (Table 3).

### 3.3. Predictors of QOL

Table 4 shows the final results of the hierarchical multiple regression models of QOL. Each block of the independent variables made a significant contribution to the variance of QOL. A total of 30.4%, and 30.8% of variance were explained by the final regression model in PCS and MCS. Results from the R^2^ change indicated that coping contributed most to the variance of MCS (17.4% of variance), while demographic characteristics contributed most to the variance of PCS (16.7% of variance). It also indicated that coping contributed second-most to the variance of PCS (11.4% of variance). Demographic characteristics, including household monthly income, chronic diseases, and unemployed time), coping (adaptive coping and maladaptive coping), and self-efficacy were strong predictors of both PCS and MCS. Moreover, adaptive coping and self-efficacy were positively associated with QOL, while maladaptive coping was negatively associated with PCS and MCS.

### 3.4. Association of Coping with QOL

**Figure 1:**
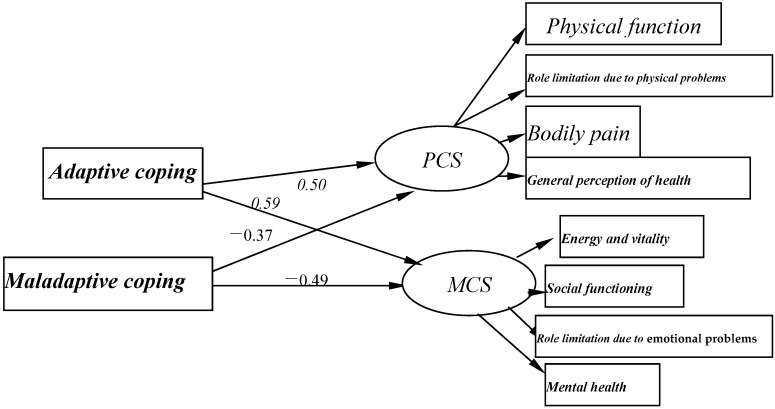
Structural equation modeling of coping and QOL (PCS and MCS).

The direct pathways of coping with QOL through self-efficacy are illustrated in Figure 1. The SEM model showed that positive associations of adaptive coping with PCS (c = 0.50, *p* < 0.01) and MCS (c = 0.59, *p* < 0.01), and significantly negative associations of maladaptive coping with PCS (c = −0.37, *p* < 0.01) and MCS (c = −0.49, *p* < 0.01) (Figure 1). This model had good fit with the data (χ^2^/df < 5, *p* < 0.01, GFI = 0.972, AGFI = 0.946, CFI = 0.972, TLI = 0.932, and RMSEA = 0.066).

### 3.5. Mediating Role of Self-Efficacy on the Associations of Coping with QOL

**Figure 2:**
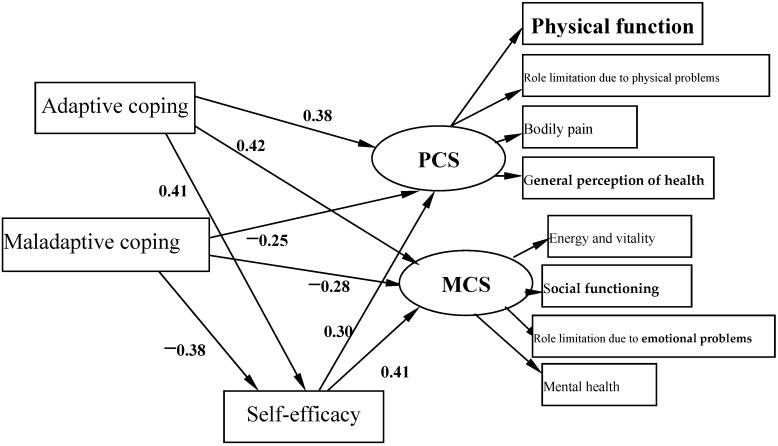
Structural equation modeling of mediating role of self-efficacy on the association between coping and QOL (PCS and MCS).

The mediating role of self-efficacy on the associations of coping with QOL is illustrated in Figure 2. Given that PCS and MCS were highly correlated with each other, the correlation between the respective residues was estimated in the model. The path coefficients of coping (adaptive coping and maladaptive coping) with PCS and MCS were decreased significantly when self-efficacy was modeled as a mediator. The bias-corrected and accelerated bootstrap test indicated that self-efficacy significantly mediated the associations of adaptive coping with PCS (a × b = 0.123, BCa 95% CI: 0.096, 0.152) and MCS (a × b = 0.167, BCa 95% CI: 0.138, 0.198). Additionally, the bias-corrected and accelerated bootstrap test indicated that self-efficacy significantly mediated the associations of maladaptive coping with PCS (a × b = −0.115, BCa 95% CI: −0.141, −0.089) and MCS (a × b = −0.156, BCa 95% CI: −0.186, −0.128). These confirmed that a significant mediating role of self-efficacy on the association between coping and QOL. The model presented in Figure 2 is fully supported by all standard goodness of fit indices (χ^2^/df < 5, RMSEMA = 0.063, CFI = 0.957, GFI = 0.972, AGFI = 0.946, and TLI = 0.933). Thus, coping directly affects PCS and MCS, and also influences PCS and MCS indirectly by the self-efficacy path.

## 4. Discussion

The results from this study indicated that most unemployed Chinese individuals suffered from impaired QOL, which were lower than the levels of the employed Chinese population [41,42,43,44,45,46] and working-age Chinese population in Sichuan urban areas [41]. These findings indicated that unemployed Chinese were significantly influenced by job loss, which resulted in experiencing low levels of both physical and mental health. These unemployed people might be vulnerable to the health-damaging effects of job loss. Furthermore, mental health was more disrupted than physical health, which supported the contention that unemployment had adverse effects on mental health and may be associated with symptoms of depression and suicide [11,47,48,49]. This may bring a new understanding to the fact that the unemployed Chinese probably faced with complicated emotions associated with job loss. Unemployment is a stressful event for individuals, and so their health may be severely impacted. In this study, physical QOL was best predicted by the demographic characteristics and coping. However, mental QOL was best predicted only by coping. Furthermore, individual personal positive resources such as self-efficacy played a mediating role in alleviating the negative effects of maladaptive coping on QOL, and promoting the positive effects of adaptive coping on QOL, which was in agreement with previous studies which concluded that positive beliefs modified the appraisal of stressful circumstances and could maintain good health [50].

In this study, demographic characteristics held the greatest importance in interpretation of physical health, accounting for 16.7% of the observed variability in PCS. Gender and age were the significant predictors of physical QOL. As for gender, females experienced slightly lower levels of physical health than males, while there was no difference in mental health between the males and females. Unemployed women may confront the loss of savings and social exclusions subsequent to job loss; as a result, they would experience a greater drive to survive. Thus, they would have to spend more time taking care of their family and undertaking a variety of domestic duties, which may result in physical health problems, and this was consistent with a previous study [51]. The older the unemployed, the worse their physical health is. Older individuals usually have lessened abilities to learn new job skills and have outdated methods of seeking reemployment. This subsequently led to a decline of involvement in job searching and introversion into oneself which, consequently, worsens their physical condition.

Low household income seemed to be also strongly and negatively associated with QOL, which was in accordance with previous studies [52,53]. The unemployed with less monthly income suffered from poorer health. In this study, 22.85% and 21.42% of the individuals had the monthly household income levels of less than 1000 Yuan (RMB) and 1000–1500 Yuan (RMB) respectively. Many families are struggling to survive due to economic difficulties brought about by unemployment, relying on minimum living subsidies to fulfill basic daily needs. This brings additional pressure to the unemployed which, in the end, could lead to poor physical and mental health.

Chronic disease was the most significant predictor of QOL. The individuals suffering from chronic diseases along with job loss usually encountered numerous negative additional impacts on their own health, as they were probably more susceptible to the adverse effects of unemployment. The individuals who suffered from a chronic disease had a significantly higher probability to lose physical strength and other detriments of their health [54]. 

In addition, individuals who had been unemployed for a longer period of time exhibited poorer physical and mental health. These findings support the link between unemployment duration and poor health [55]. Winefield found that the damage to mental well-being and the intensity of psychological distress depended greatly on the duration of unemployment [56]. The long unemployment periods caused individuals to lose confidence in possible re-employment and working skills. They became more passive, lacked motivation, and were unwilling to change their situations. The stress would be accumulated and their worries and tension would be escalated as unemployment extended and savings exhausted. As time goes by, the damage to mental health would become more severe. Subsequently, they were more vulnerable to perceiving the damage of unemployment on their physical health. Previous studies suggested that as the length of unemployment increased, there was increased urinary catecholamine reactivity to uncontrollable feedback, reduced persistence on task, and they became more concerned with external causes than internal skills [57]. The long unemployment duration can cause profound deleterious physical and psychological health effects, which are in agreement with previous findings that showed long-term unemployed persons had more depressive episodes in the past 12 months as compared to the short-term unemployed [58]. Extended periods of unemployment are harmful to QOL of unemployed people. 

However, the extent of perceived QOL does not only rely on the demographic characteristics, but also considerably relies on coping. In this study, the transactional stress model theory was expanded to test the associations of coping, self-efficacy, and QOL. This model indicated that an individual’s reaction to stress could be influenced by the process of coping with an event that has been appraised as stressful, and coping are affected by personal resources, like self-efficacy, which has yielded various findings in regards to individuals’ health [26]. As hypothesized, this study found a significant relationship between coping and QOL. Coping contributed most to both physical and mental health, accounting for 11.4% and 17.4% of the total variance. Additionally, coping had strong total effects on PCS and MCS (Figure 1). This indicated that coping could be utilized to contend with unemployment and maintain optimal QOL. Adaptive coping, such as acceptance, planning, and positive reframing, are the kind of problem-focused coping strategies [59], and help individuals to adapt to the stressful situation, takes active actions to cope with the stress, allows an individual greater perceived control over their problems, can ameliorate the stress and maintain their well-being, and is positively associated with physical and mental health. Maladaptive coping, such as denial, self-blame, substance use, and religion, are the emotion-focused coping strategies [60], which may sometimes lead to a reduction in perceived control. Previous study shows that some emotion-focused coping strategies, e.g., self-blame, have been associated with anxiety and depression, as well as psychosomatic complaints and psychological stress actions [61]. The unemployed with maladaptive coping are likely passively and negatively to appraise the stress and could not deal well with the job loss. They might avert their attention to distracting activities, such as substance use, which may result in the adverse effects of unemployment on health. Thus, coping could facilitate adjustment to job loss and protect the adverse effects of unemployment on QOL. This finding contributes to the understanding that today’s unemployed need to draw from heretofore unrecognized, and largely untapped, positive resources, such as positive coping abilities, to help them combat the dysfunctional effects of job loss.

The study findings also indicated that self-efficacy mediated the effects of coping on QOL, which was in support with previous studies [62] that coping was highly correlated with personal resources, like self-efficacy, and promoted health and well-being. Individuals who perceived more adaptive coping might be more likely to develop a high sense of self-efficacy, which resulted in their high possibilities to perceive challenges as surmountable; thus, their anxiety and worries would be decreased. Those people would control bad emotions and strive for the positive aspects. Subsequently, they would be more motivated to find a way to solve the problem, and more actively process the information of the outside world; thus, they are more likely to adapt to the stressful situation. Consequently, their health will not be severely affected. This, in turn, positively impacted their QOL, whereas, individuals who perceived more maladaptive coping might have a lower sense of self-efficacy, and would be easily convinced that their efforts to address difficult challenges were futile, thus being less able to dealing reasonably with unemployment. They would consider themselves to be inadequate to face the task, and so the problem would seem insurmountable. This will generate psychological pressure which, in most cases, causes the individuals to concentrate on possible failures and adverse consequences, rather than to consider how to effectively use their abilities to achieve the goals. Furthermore, low self-efficacy was also related to vulnerability to stress and psychological distress [63]. Self-efficacy is considered a crucial determinant of emotional reactions to the stressful situation, and it plays a partial mediating role on the association between coping and QOL. Positive psychological resources, one of which is self-efficacy, have a particularly marked effect on how well individuals can deal with unemployment: the person involved copes with the loss of his/her job more successfully if he/she enjoys high self-efficacy. The individuals’ coping abilities not only directly affected both physical and mental health, but also indirectly affected QOL through the path of self-efficacy. This result implies that developing skills of active coping should result in a higher sense of self-efficacy which, in turn, may help employees to combat stress, and reduce impairments of unemployment and maintain optimal QOL. Investment in self-efficacy and adaptive coping may be mostly effective in reducing the effects of unemployment, and improving the perceived quality of life. Armed with the implications of this empirical evidence, we propose that human resource training and development efforts recognize and enhance the underemphasized positive resources.

The present study bears the limitations that it is characterized by cross-sectional research, therefore, one cannot derive any conclusion on the causality of the associations observed between unemployment and QOL. Additionally, unemployed individuals were selected by convenience sampling, which may limit the generalizability of this study to other populations. However, despite the above limitations, this study has notable strong points. Firstly, the sample size was quite large. Secondly, unemployed individuals were selected from 23 cities in eastern, central, and western regions of China with a wide coverage of the country, which might indicate the health status of the unemployed in the whole country. Finally, there was a high response rate, most likely due to the fact that face-to-face interviews allowed for greater collection of information.

## 5. Conclusions

Unemployed Chinese people suffered from more impaired mental QOL than physical QOL. Investment in coping could improve QOL by promoting better management of issues brought about by unemployment. In addition, self-efficacy has the ability to reduce the impact of unemployment on QOL, by mediating coping. This study highlights the need of coping for better management of unemployment in order to improve QOL, and the enhancement of self-efficacy to improve coping abilities. Specifically, we propose that human resource development strategies aim at enhancing the components of employees’ positive coping or self-efficacy to reduce their perceptions of the adverse effects of unemployment on QOL.

## Figures and Tables

**Table 1:** Demographic characteristics of unemployed people (*N* = 1825).

Variables	Number (*N*)	Percent (%)
**Gender**		
Male	1022	56.00
Female	803	44.00
**Age**		
<25	367	20.11
25–39	806	44.16
>39	652	35.73
**Marital status**		
Married	1073	58.79
Others	752	41.21
**Educational level**		
≤senior high school	1156	63.34
>senior high school	669	36.66
**Household monthly income (Yuan)**		
≤1000	417	22.85
1001–1500	391	21.42
1501–2000	420	23.01
>2000	597	32.71
**Chronic diseases**		
Yes	994	54.47
No	831	45.53
**Unemployed time (months)**		
1–2	387	21.21
3–5	372	20.38
6–11	454	24.88
≥12	612	33.53
	**Mean ± S.D.**	
Physical function	84.3 ± 19.7	
Role limitation due to physical problems	65.4 ± 33.3	
Bodily pain	72.9 ± 22.0	
General perception of health	61.4 ± 18.4	
Energy and vitality	61.0 ± 17.8	
Social functioning	70.1 ± 20.4	
Role limitation due to emotional problems	58.4 ± 36.6	
Mental health	59.6 ± 17.6	

**Table 2:** QOL scores of unemployed people on demographic characteristics (*N* = 1825).

Variables	PCS	MCS
**Gender**		
Male	71.8 ± 17.6	62.9 ± 17.1
Female	70.2 ± 17.2 *	61.9 ± 16.5
**Age**		
<25	77.6 ± 14.8 **	64.5 ± 15.6 *
25–39	71.4 ± 16.7 *	61.7 ± 16.2
>39	66.9 ± 18.5	62.1 ± 18.3
**Marital status**		
Married	69.2 ± 17.4 **	62.5 ± 17.2
Others	73.8 ± 16.3	62.3 ± 15.9
**Education level**		
≤senior high school	69.6 ± 17.3 **	62.1 ± 17.0
>senior high school	73.6 ± 16.4	63.1 ± 16.3
**Household monthly income (Yuan)**		
≤1000	67.0 ± 18.1 **	58.7 ± 17.1 **
1001–1500	70.3 ± 17.3 *	61.5 ± 16.8 *
1501–2000	71.0 ± 17.5 *	63.1 ± 16.3
>2000	74.4 ± 16.4	65.1 ± 16.7
**Chronic diseases**		
Yes	64.0 ± 17.3 **	59.2 ± 16.8 **
No	76.8 ± 15.4	65.0 ± 16.5
**Unemployed time**		
1–2 month	76.2 ± 15.7	65.1 ± 16.8
3–5month	73.8 ± 16.0 *	63.8 ± 15.2 *
6–11 month	70.2 ± 17.5 **	62.0 ± 15.6 *
≥12 month	66.9 ± 18.2 **	60.0 ± 18.4 **

* *p* < 0.05; ** *p* < 0.01.

**Table 3:** Correlation of PCS and MCS and related factors.

	Mean	SD	PCS	MCS	Adaptive Coping	Maladaptive Coping	Self-Efficacy
**PCS**	71.1	17.4	1				
**MCS**	62.4	16.9	0.620 **	1			
**Adaptive coping**	45.7	7.2	0.275 **	0293 **	1		
**Maladaptive coping**	40.6	6.9	−0.140 **	−0.193 **	0.400 **	1	
**Self-efficacy**	38.3	5.7	0.332 **	0.450 **	0.259 **	−0.220 **	1

** *p* < 0.01.

**Table 4:** The hierarchical multiple regression models of PCS and MCS.

Variables	PCS			MCS		
	Model 1 b(B)	Model 2 b(B)	Model 3 b(B)	Model 1 b(B)	Model 2 b(В)	Model 3 b(B)
**Intercept**	77.334 **	61.775 **	42.673 **	73.377 **	61.306 **	28.244 **
**Block 1 Demographic characteristics**						
**Gender**	−1.800 * (−0.051)	−1.891 * (−0.054)	−1.541 * (−0.044)	−1.289 (−0.038)	−1.366 (−0.040)	−0.822 (−0.024)
**Age**						
25–39 vs. <25	−3.287 ** (−0.094)	−1.574 (−0.045)	−1.630 (−0.046)	−2.437 * (−0.072)	−0.493 (−0.015)	−0.454 (−0.013)
>39 vs. <25	−2.615 (−0.072)	−1.182 (−0.032)	−1.375 (−0.038)	0.414 (0.012)	2.336 (0.066)	1.974 (0.056)
**Marital status**	−0.045 (−0.001)	0.687 (0.019)	0.756 (0.021)	−2.486 * (−0.073)	−1.628 (−0.048)	−1.449 (−0.042)
**Educational level**	0.847 (0.023)	0.609 (0.017)	0.347 (0.010)	−0.251 (−0.007)	−0.564 (−0.016)	−1.027 (−0.029)
**Chronic diseases**	−10.876 ** (−0.311)	−10.159 ** (−0.290)	−9.891 ** (−0.282)	−5.737 ** (−0.170)	−5.049 ** (−0.149)	−4.562 ** (−0.135)
**Household monthly income (yuan)**						
1001–1500 vs. ≤1000	2.300 (0.054)	2.295 * (0.054)	2.332 * (0.055)	2.386 * (0.058)	2.252 * (0.055)	2.403 * (0.059)
1501–2000 vs. ≤1000	3.043 ** (0.074)	2.100 (0.051)	2.211 * (0.053)	4.005 ** (0.100)	2.718 * (0.068)	3.016 ** (0.076)
>2000 vs. ≤1000	5.031 ** (0.136)	3.627 ** (0.098)	3.116 ** (0.084)	5.206 ** (0.145)	3.211 ** (0.089)	2.476 * (0.069)
**Unemployed time (months)**						
3–5 vs. 1–2	−0.317 (−0.007)	0.474 (0.011)	0.698 (0.016)	−0.698 (−0.017)	0.415 (0.010)	0.771 (0.019)
6–11 vs. 1–2	−2.487 * (−0.061)	−1.567 (−0.039)	−1.256 (−0.031)	−1.973 (−0.050)	−0.844 (−0.022)	−0.258 (−0.007)
≥12 vs. 1–2	−3.763 ** (−0.102)	−3.541 ** (−0.096)	−3.283 ** (−0.089)	−3.807 ** (−0.107)	−3.576 ** (−0.100)	−3.009 ** (−0.084)
**Block 2 Coping**						
Adaptive coping		0.856 ** (0.344)	0.684 ** (0.275)		0.973 ** (0.407)	0.682 ** (0.285)
Maladaptive coping		−0.639 ** (−0.248)	−0.473 ** (−0.184)		−0.860 ** (−0.348)	−0.573 ** (−0.232)
**Block 3 Self-efficacy**			0.531 ** (0.174)			0.906 ** (0.306)
**R^2^**	0.167	0.281	0.304	0.061	0.235	0.308
**△R^2^**	0.167	0.114	0.023	0.061	0.174	0.073

* *p* < 0.05; ** *p* < 0.01.

## Abbreviations

The following abbreviations are used in this manuscript:

QOL	Quality of life
SF-36	the 36-item Short-Form Health Survey
PCS	physical component summary
MCS	mental component summary
ANOVA	one-way analysis of variance
HMR	Hierarchical multiple regression
SEM	Structural equation model
